# Comparison of the Coincidence of Osteoporosis, Fracture, Arthritis Histories, and DEXA T-Score between Monozygotic and Dizygotic Twins: A Cross-Sectional Study Using KoGES HTS Data

**DOI:** 10.3390/nu14183836

**Published:** 2022-09-16

**Authors:** Hyo Geun Choi, So Young Kim, Bong Cheol Kwon, Ho Suk Kang, Hyun Lim, Joo-Hee Kim, Ji Hee Kim, Seong Jin Cho, Eun Sook Nam, Kyueng Whan Min, Ha Young Park, Nan Young Kim, Younghee Choi, Mi Jung Kwon

**Affiliations:** 1 Department of Otorhinolaryngology-Head & Neck Surgery, Hallym University Sacred Heart Hospital, College of Medicine, Hallym University, Anyang 14068, Korea; 2 Department of Otorhinolaryngology-Head & Neck Surgery, CHA Bundang Medical Center, College of Medicine, CHA University, Seongnam 13488, Korea; 3 Department of Orthopedic Surgery, Hallym University Sacred Heart Hospital, College of Medicine, Hallym University, Anyang 14068, Korea; 4 Division of Gastroenterology, Department of Internal Medicine, Hallym University Sacred Heart Hospital, College of Medicine, Hallym University, Anyang 14068, Korea; 5 Division of Pulmonary, Allergy, and Critical Care Medicine, Department of Medicine, Hallym University Sacred Heart Hospital, College of Medicine, Hallym University, Anyang 14068, Korea; 6 Department of Neurosurgery, Hallym University Sacred Heart Hospital, College of Medicine, Hallym University, Anyang 14068, Korea; 7 Department of Pathology, Kangdong Sacred Heart Hospital, College of Medicine, Hallym University, Seoul 05355, Korea; 8 Department of Pathology, Hanyang University Guri Hospital, College of Medicine, Hanyang University, Guri 11923, Korea; 9 Department of Pathology, Busan Paik Hospital, College of Medicine, Inje University, Busan 47392, Korea; 10 Hallym Institute of Translational Genomics and Bioinformatics, Medical Center, Hallym University, Anyang 14068, Korea; 11 Department of Pathology, Hallym University Dongtan Sacred Heart Hospital, College of Medicine, Hallym University, Hwaseong 18450, Korea; 12 Research Institute for Complementary & Alternative Medicine, Hallym University, Anyang 14068, Korea; 13 Department of Pathology, Hallym University Sacred Heart Hospital, College of Medicine, Hallym University, Anyang 14068, Korea

**Keywords:** dizygotic twins, monozygotic twins, aging, bone mineral density, osteoporosis, fracture, osteoarthritis, rheumatoid arthritis, genetic factors, environmental factors

## Abstract

We explored the genetic and environmental inter-relationships among osteoporosis, fracture, arthritis, and bone mineral density concordance in monozygotic twins compared to those in dizygotic twins. This cross-sectional research assessed data of 1032 monozygotic and 242 dizygotic twin pairs aged >20 years included in the Healthy Twin Study data of the Korean Genome and Epidemiology Study between 2005 and 2014. Outcomes of interest included illness concordance and absolute differences in dual-energy X-ray absorptiometry (DEXA) T-scores. We found comparable concordances of osteoporosis, fractures, osteoarthritis, and rheumatoid arthritis between monozygotic and dizygotic twins. Medical histories of osteoporosis, fractures caused by accident or falling, osteoarthritis, and rheumatoid arthritis were not distinct between monozygotic and dizygotic twins. Accidental fracture occurrence in both monozygotic twins showed significantly lower odds than that in dizygotic twins. Genetic influence on liability to fracture risk might thus be maintained. DEXA T-scores for bone mineral density indicated more comparable tendencies within monozygotic twin pairs than within dizygotic ones, suggesting the relative importance of genetic contribution to bone mineral density. The relative importance of genetic factors in bone mineral density is sustained between monozygotic twins; overt disease expression of osteoporosis, fractures, or arthritis may be affected by environmental factors.

## 1. Introduction

Osteoporosis, fractures, osteoarthritis (OA), and rheumatoid arthritis (RA) are highly prevalent diseases that are associated with considerable morbidity, mortality, and healthcare costs [1,2,3,4,5]. Age-related bone and joint diseases are closely interrelated and characterized by multifaceted interactions between genetic and environmental factors [6,7,8,9]. While aging is one of the main risk factors for the development of osteoporosis, fractures, OA, and RA [9,10,11], other factors such as genetic predisposition, obesity, inflammation, sex and hormones, or metabolic syndrome contribute to the development of these diseases [8,12,13,14].

Osteoporosis is characterized by a reduction in bone mineral density and quality, with a consequent increase in bone fragility and risk of fracture [15,16]. Updates to the epidemiology and pathophysiology of osteoporosis have proposed an inverse relationship with OA and a positive correlation with RA [17]: higher bone mineral density and lower fracture risk in patients with OA [2,3], and reduced bone mineral density and high frequency of osteoporosis in patients with RA compared with those in the general population [1]. In other rheumatic diseases such as ankylosing spondylitis, a high prevalence of bone mineral density and osteoporosis is observed in patients with early-stage disease [18]. In this context, subclinical radiologic changes related to bone mineral density might be a clinically important intermediate process in overt diseases, in which a potential indicator for disease expression might be expected.

Twin studies may offer a valuable approach for estimating the relative significance of genetic and environmental contributions to these bone and joint diseases and complex traits, given the genetic similarity and shared family and rearing environment between twins [19,20]. A higher correspondence between monozygotic twin pairs than between dizygotic ones may be ascribed to genetic factors, while a comparable degree of similitude between monozygotic and dizygotic twin groups may be attributed to an environmental influence [19]. A genetic predisposition to age-related bone diseases was suggested in individual epidemiologic studies, including a few twin studies, with the possible individual heritability of bone mineral density (60–80%) [21], osteoporosis (36–85%) [22,23], fracture (68%) [7,8], falls (35%) [24], frailty (43%) [25], OA (39–65%) [14], and RA (52–60%) [26,27,28]. However, combined analyses of aging-related bone and joint diseases and their relevance to bone mineral density, with full adjustments for lifestyle factors, have not been determined in validated twin studies. Since osteoporosis, fractures, OA, and RA appear to share possible risk factors and reciprocal associations, further studies adjusting for possible mutual confounders are needed.

This study aimed to evaluate the putative effects of environmental factors on the genetic predisposition toward aging-related bone disease by comparing twin cohorts. To explore this issue, we investigated medical histories and concordances of aging-related bone diseases and absolute differences in dual-energy X-ray absorptiometry (DEXA) T-scores regarding bone mineral density between monozygotic twins (genetic influence) and dizygotic twins (environmental contribution over genetic influence) after adjusting for lifestyle factors.

## 2. Materials and Methods

### 2.1. Study Population and Data Collection

The ethics committee of Hallym University (2021-03-004) approved the use of the data. The requirement for written informed consent was waived by the institutional review board. This cohort study was based on the Healthy Twin Study (HTS), a nationwide cross-sectional survey that is a part of the prospective Korean Genome Epidemiology Study (KoGES), that recruited Korean same-sex twin pairs aged over 20 years who primarily resided in Seoul or Busan, the two largest urban areas in Korea [29]. The participants were voluntarily recruited through advertisements and media at health-related governmental agencies and participating hospitals since 2005 [30]. The baseline data of KoGES HTS were obtained from 2005 to 2013 and follow-up data from 2008 to 2014. Zygosity was assessed at baseline using genetic analysis, including 16 short tandem repeat markers (Amp*Fl*STR Identifier Kit; Perkin Elmer, Waltham, MA, USA) [31]. Two-thirds of the participants who completed the baseline examination were followed up, and their medical histories were updated. The study data were described in detail in previous studies [29,30,32,33].

### 2.2. Participants Selection

**Figure 1:**
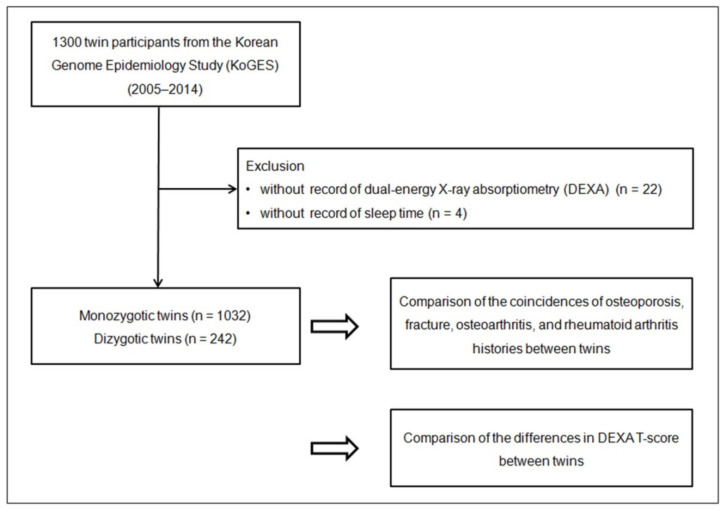
The study design of the present study; 1032 monozygotic twins and 242 dizygotic twins were compared for concordance of several aging-related bone and joint diseases, including osteoporosis, fracture, osteoarthritis, and rheumatoid arthritis between twins. DEXA T-scores for bone mineral density were also compared.

Among 1300 twin participants, those who did not have the records of DEXA data measuring bone mineral density (*n* = 22) and sleep time (*n* = 4) were excluded. A total of 1032 monozygotic twins (516 pairs of twins) and 242 dizygotic twins (121 pairs of twins) were enrolled (Figure 1). We then analyzed the concordance of their disease histories and DEXA T-scores between the monozygotic and dizygotic twin participants.

### 2.3. Survey

Participants completed the interviewer-administered KoGES Baseline Core Questionnaire officially designed by the Korean National Institute of Health to collect information on demographic characteristics and lifestyle, including dietary habits, health conditions, and medical history; the questionnaire is publicly available on the Korean National Institute of Health website (https://nih.go.kr/contents.es?mid=a50401010300) (accessed on 1 January 2022). Face-to-face interviews by trained interviewers were performed to clarify incomplete or ambiguous responses [29,34]. The listed questionnaires and data are regularly validated and updated. Interviewer-administered questionnaires, anthropometric measurements, and biochemical tests were conducted at the officially designated University hospitals and medical institutions, as previously described in detail [29].

Trained interviewers asked the participants about their medical history of osteoporosis, fracture by accident, fracture by fall, OA, or RA. Bone mineral density was measured using dual-energy DEXA (Lunar Radiation, Madison, WI, USA; and Delphi W; Hologic, Boston, MA, USA) for the whole body [20]. We used the mean bone mineral density of the whole body. All twins underwent bone mineral density measurements using the same DEXA machine at the same center. These devices were maintained using the standard quality control procedures recommended by the manufacturer to assure that the calibration of bone mineral density remained constant, and the coefficients of variation for bone mineral density measurement were 1.0% for the two machines. The results were expressed as T-scores. The T-score is a measure of deviation from the expected population mean value of the peak young adult bone mass [13]. Clinically, it is used to predict fracture risk. All DEXA data were expressed as mean ± standard deviation (SD).

The income group was divided into low income (<USD2000 per month), middle income (~USD2000–USD3999 per month), and high income (≥USD4000 per month) groups based on household income. Education was grouped as under high school, graduated from high school, dropped out of college, or graduated from college. Marital status was assessed as unmarried, married, divorced, or other. Physical activity levels were assessed using hard, moderate, walking, and sitting times. Physical activity was measured both at home and at the workplace. Body mass index was calculated as kg/m^2^ using the health checkup data. Smoking history was classified as nonsmoker (<100 cigarettes in entire life), past smoker (quit more than 1 year ago), and current smoker. Drinking alcohol habits were categorized as non-drinkers, ≤1 time per month, 2–4 times per month, and ≥2 times per week. Sleep time was calculated as 5/7 weekdays plus 2/7 weekends.

### 2.4. Exposure

Monozygotic and dizygotic twins were considered independent variables in this study. All the participants were twins. There were no triplets, quadruplets, or higher-order multiples.

### 2.5. Outcomes

We calculated the coincidence of medical histories between matched twin participants. These data were categorized as positive–positive, positive–negative, or negative–negative.

Additionally, we calculated the absolute difference in the DEXA T-scores between the matched twin participants. For example, one of the twin participants had a T-score of 2, and the other had a T-score of 1; thus, the absolute difference in T-score was calculated as 1.

### 2.6. Statistical Analyses

A Chi-square test (categorical variables) or Wilcoxon rank sum test (continuous variables) was performed to compare the general characteristics of the participants between different patient groups.

We calculated the odds ratios (OR) with 95% confidence intervals (CI) of the coincidence of disease histories. First, we calculated the OR of monozygotic twins ([positive–positive or negative–negative]/[positive–negative]) compared to that of dizygotic twins using a binomial logistic regression model. Second, we calculated the OR of monozygotic twins ([positive–positive]/[positive–negative]/[negative–negative]/) compared with that of dizygotic twins using a multinomial logistic regression model.

We calculated the estimated values (EV) with a 95% CI of the absolute difference in the T-score. EV was measured as the absolute difference between monozygotic twins minus the absolute difference between dizygotic twins, using a linear regression model.

Crude, adjusted model 1 (age, sex, income, education, marital status, physical activity, obesity, smoking habit, frequency of alcohol consumption, and sleep time) and adjusted model 2 (model 1 plus history of each disease [osteoporosis, fracture by accident, fracture by falling, OA, and RA]) were used.

Two-tailed analyses were conducted and a *p*-value < 0.05 was considered statistically significant. The results were statistically analyzed using SPSS software (version 24.0; IBM, Armonk, NY, USA).

## 3. Results

The baseline features of the monozygotic and dizygotic twins are summarized in Table 1. The medical histories of osteoporosis, fractures by accident or falling, OA, and RA or DEXA T-scores were not dissimilar between monozygotic and dizygotic twins (all *p* > 0.05). The distribution of age groups, sex ratio, and hard-level physical activity showed differences between the two groups (*p* = 0.003, *p* = 0.021, and *p* = 0.019, respectively). Other components of household income, education, marriage, overall physical activity except for hard level, obesity, smoking, alcohol consumption, and sleeping hours did not differ between monozygotic and dizygotic twins (all *p* > 0.05).

With full adjustment analyses, we determined any possible associations of the concordance rates in terms of the presence or absence of osteoporosis, fractures by accident or by falling down, OA, and RA within monozygotic compared to those within dizygotic twins (Table 2). Crude or adjusted ORs for concordances of these diseases within monozygotic twins were not significantly higher than those within dizygotic twins (all *p* > 0.05). However, we observed a trend toward an increased likelihood of concordant association of fracture by falling in monozygotic twins with borderline significance (adjusted OR, 1.44 [95% CI, 0.97–2.15]; *p* = 0.070).

We further investigated whether the occurrence of at least one incident disease (in terms of osteoporosis, fractures by an accident or falling down, OA, or RA) in monozygotic twin pairs was more frequent than that in dizygotic twin pairs (Table 3). The occurrence of fractures by accident in both monozygotic twin pairs showed lower odds than in dizygotic twin pairs after full adjustment (adjusted OR 0.25 [95% CI, 0.08–0.79]; *p* = 0.018). The occurrence of at least one other bone disease was not higher in monozygotic than in dizygotic twins (all *p* > 0.05).

We calculated differences in DEXA T-scores between the matched monozygotic and dizygotic twin pairs to analyze the EV (Table 4). The difference in the DEXA T-score was significantly higher within dizygotic than within monozygotic twins (EV, 0.62 [95% CI, 0.45–0.79]; *p* <0.001), indicating a similar tendency within monozygotic twins.

## 4. Discussion

In this cross-sectional study based on validated twin cohorts, we could not identify any significant concordances of age-related bone and joint diseases or any increased likelihood of osteoporosis, fractures, OA, or RA within monozygotic twins compared to those within dizygotic twins through concurrent adjustments for comprehensive confounding (e.g., lifestyle and socioeconomic) factors. Nonetheless, the relationship between DEXA T-scores and bone density changes indicated a highly comparable trend within monozygotic twins compared to that within dizygotic twin pairs. Owing to the rarity of qualified twin cohort data, our current epidemiological study might promote the understanding of the environmental influence on genetic contribution in overt osteoporosis, fractures, OA, or RA, which are critical global issues and a healthcare burden in an aging society.

The combined analysis of osteoporosis, fractures, OA, and RA with full adjustments for lifestyle and environmental factors has not been conducted in twin studies. We found that there were no higher concordances of incident osteoporosis or fractures, OA, or RA within monozygotic twins compared to those within dizygotic twins. However, a trend toward concordant association of fracture by falling in monozygotic twins with borderline significance was observed: the occurrence of fractures by accident in both monozygotic twin pairs showed lower odds than that in dizygotic twin pairs. Because of the relatively minor excess in concordance in monozygotic twins compared with that in dizygotic twins, these findings might carefully indicate the low genetic influence on liability to fracture risk, consistent with a previous twin study [22,35]. Anatomically identical stress fractures are repeatedly described in monozygotic twins [35,36,37]. However, the indistinct or vague concordance in the phenotype of these diseases within monozygotic twins in the present study might be attributed to the possible contribution of environmental factors relevant to dissimilar acquired lifestyle behaviors between monozygotic twin pairs who share an identical genetic background, sex, and age [9]. Molecular studies, including epigenome-wide association studies, might provide one of the possible explanations for the discordant disease expressions in monozygotic twins, indicating the complex interplay between genetic, environmental, and epigenetic influences on osteoporosis, fractures, OA, and RA [12,38,39]. While twins are epigenetically indistinguishable during the early phase of life, older monozygotic twins harbor prominent disparities in the genomic distribution of 5-methylcytosine DNA and histone acetylation [7], which might lead to differential gene expression signatures and disease phenotypes with age [40]. Similarly, one prospective study on Finnish twins indicated that environmental factors are more likely to make an adult population susceptible to fractures [23]. Genetic variations related to osteoporosis and fracture may depend on fracture type and age [41]. The overall age-adjusted fracture accounted for a genetic contribution of <20% [41]. Since exclusively adult populations were included in this study, different environmental or acquired influences might lessen the concordance likelihood of overt bone and joint diseases in monozygotic twin pairs compared to those in dizygotic twin pairs.

Although bone mineral density is associated with the risk of osteoporosis, fractures, OA, and RA, a comparative study using the DEXA T-score in these bone and joint diseases has not been assessed in twin studies. The clinical significance of baseline radiologic parameters, such as the DEXA T-score, appears to be underestimated in the evaluation of genetic contribution when comparing twin pairs. In our study, despite the discordance of disease expression within monozygotic twins, we have shown a highly similar tendency of DEXA-T-scores between monozygotic twins compared to that between dizygotic twins (EV = 0.62, 95% CI, 0.45–0.79, *p* < 0.001), which seems to suggest the relative importance of genetic contribution in bone mineral density at the subclinical level over environmental factors. Our findings support the results of a previous Korean twin study indicating the high heritability of bone mineral density [20]. Furthermore, the present results are also in line with those of several European population-twin studies that have shown fewer differences in bone mass between monozygotic and dizygotic twins, suggesting that there may be substantial heritability (50–85%) and genetic traits for bone mineral density [26,27]. Genome-wide association studies have revealed several genetic polymorphisms associated with bone mineral density, osteoporosis, and fracture [8]. However, these genotype determinations have failed to identify individuals at an increased risk of osteoporosis [42], which might imply additional triggers mediated by extrinsic factors. Gene–environment interactions, such as smoking, alcohol consumption, diet, physical activity, and epigenetic differences, might result in discordance in disease expression between monozygotic twin pairs [27]. Thus, the relative genetic and environmental significance of bone mineral density using DEXA T-scores seems to be comparable with those of previous twin studies mainly based on European populations [26,27].

The strength of this study was based on prospective twin cohort data with follow-up data composed of both monozygotic and dizygotic twins, KoGES HTS, with qualified data quality regularly validated by national statisticians, which made our findings more reliable. We comprehensively considered potential confounders of lifestyle factors, including physical activity, obesity, smoking, alcohol consumption, sleep duration, and socioeconomic status, including income level, education level, and marital status, comparing twin pairs. Adjustments for lifestyle factors (income, alcohol consumption, smoking status, physical activity, marital status, and obesity) may be additional strengths because they were listed as relevant risk factors for bone and joint diseases [43]. To the best of our knowledge, this study is the first combined analysis covering osteoporosis, fracture, OA, and RA with full adjustment for comprehensive lifestyle and socioeconomic factors.

Our study has some limitations that should be addressed. First, even though substantial variables were adjusted in this study, unmeasured confounders may remain, and they could not be completely excluded. Second, the causal relationship between twins and bone and joint diseases could not be confirmed by a cross-sectional study design. Third, the relatively small number of concordant diseases between both twin pairs may be limited in this study, despite a large number of twin participants with bone and joint diseases. Fourth, the absence of genetic data on related bone and joint diseases or no information on diet may be another limitation. Finally, we did not calculate the bone mineral density from the thoracic and lumbar spine, which is a common site of fractures due to osteoporosis.

## 5. Conclusions

In conclusion, while the relative importance of genetic factors in bone mineral density may be sustained between monozygotic twins, the disease expression of osteoporosis, fractures, OA, or RA might be affected by environmental factors. Our results provide supportive evidence that common bone and joint diseases may be preventable.

## Figures and Tables

**Table 1:** Baseline features of the monozygotic and dizygotic twins.

Characteristics		Total Participants		
		Monozygotic Twins	Dizygotic Twins	*p*-Value
Age (years, *n*, %)				0.003 *
	20–24	6 (0.6)	0 (0)	
	25–29	64 (6.2)	4 (1.7)	
	30–34	352 (34.1)	85 (35.1)	
	35–39	242 (23.4)	65 (26.9)	
	40–44	139 (13.5)	36 (14.9)	
	45–49	129 (12.5)	20 (8.3)	
	50–54	82 (7.9)	22 (9.1)	
	55–59	12 (1.2)	10 (4.1)	
	60–64	4 (0.4)	0 (0)	
	65+	2 (0.2)	0 (0)	
Sex (n, %)				0.021 *
	Males	378 (36.6)	108 (44.6)	
	Females	654 (63.4)	134 (55.4)	
Income (n, %)				0.959
	<2 million (won)	342 (33.1)	81 (33.5)	
	2 to <3 million (won)	276 (26.7)	68 (28.1)	
	3 to <4 million (won)	210 (20.3)	48 (19.8)	
	≥4 million (won)	204 (19.8)	45 (18.6)	
Education (n, %)				0.798
	Under high school	119 (11.5)	25 (10.3)	
	Graduated from high school	365 (35.4)	90 (37.2)	
	Commercial college—Dropped out of college	121 (11.7)	32 (13.2)	
	Graduated from college	427 (41.4)	95 (39.3)	
Marriage (n, %)				0.362
	Unmarried	242 (23.4)	50 (20.7)	
	Married	723 (70.1)	171 (70.7)	
	Divorced or others	67 (6.5)	21 (8.7)	
Physical Activity				
	Hard (hour/1 week, mean, SD)	3.1 (6.8)	4.6 (9.7)	0.019 *
	Moderate (hour/1 week, mean, SD)	5.8 (10.5)	6.2 (10.2)	0.666
	Walk (hour/1 week, mean, SD)	6.1 (9.6)	6.9 (10.9)	0.287
	Sit (hour/1 week, mean, SD)	40.2 (22)	37.8 (20.8)	0.118
Obesity (n, %)				0.232
	Underweight (BMI < 18.5)	27 (2.6)	5 (2.1)	
	Normal (BMI ≥ 18.5 to <23)	497 (48.2)	112 (46.3)	
	Overweight (BMI 23 to <25)	217 (21)	67 (27.7)	
	Obese I (BMI ≥ 25 to <30)	259 (25.1)	52 (21.5)	
	Obese II (BMI ≥ 30)	32 (3.1)	6 (2.5)	
Smoking status (n, %)				0.172
	Nonsmoker	678 (65.7)	145 (59.9)	
	Past smoker	106 (10.3)	33 (13.6)	
	Current smoker	248 (24)	64 (26.4)	
Frequency of drinking alcohol (n, %)				0.360
	Nondrinker	302 (29.3)	64 (26.4)	
	≤1 time per month	232 (22.5)	46 (19)	
	2–4 times per month	295 (28.6)	79 (32.6)	
	≥2 times per week	203 (19.7)	53 (21.9)	
Sleeping hours (n, %)				0.367
	≤5 h	53 (5.1)	16 (6.6)	
	6–7 h	610 (58.7)	146 (59.8)	
	8–9 h	349 (33.6)	72 (29.5)	
	≥10 h	28 (2.7)	10 (4.1)	
Osteoporosis (n, %)		27 (2.6)	4 (1.7)	0.491
Fracture by accident (n, %)		80 (7.8)	25 (10.3)	0.189
Fracture by falling down (n, %)		128 (12.4)	23 (9.5)	0.226
Osteoarthritis (n, %)		37 (3.6)	7 (2.9)	0.699
Rheumatoid arthritis (n, %)		20 (1.9)	3 (1.2)	0.598
DEXA T-score (mean, SD)		0 (1.6)	0 (1.8)	0.932

* Significance at *p* < 0.05. Chi-square test (categorical variables) or Wilcoxon rank sum test (continuous variables) was performed. Abbreviation: BMI, body mass index; SD, standard deviation; DEXA, dual-energy X-ray absorptiometry.

**Table 2:** Analysis of odds ratios with 95% confidence interval of coincidence of bone diseases in monozygotic twins compared to that in dizygotic twins (reference: positive/negative of diseases between twins).

Coincidence of Diseases	Monozygotic Twins	Dizygotic Twins	Odds Ratios (95% Confidence Interval)					
	*n* (%)	*n* (%)	Crude	*p*	Model 1 *	*p*	Model 2 †	*p*
Osteoporosis								
Concordant	998/1032 (96.7)	238/242 (98.3)	2.03 (0.71–5.77)	0.185	1.97 (0.60–6.41)	0.261	1.99 (0.60–6.58)	0.263
Discordant	34/1032 (3.3)	4/242 (1.7)	1		1		1	
Fracture by accident								
Concordant	888/1032 (86)	204/242 (84.3)	1.15 (0.78–1.69)	0.484	1.14 (0.74–1.75)	0.563	1.09 (0.71–1.68)	0.688
Discordant	144/1032 (14)	38/242 (15.7)	1		1			
Fracture by falling down								
Concordant	820/1032 (79.5)	204/242 (84.3)	1.39 (0.95–2.03)	0.089	1.45 (0.98–2.15)	0.066	1.44 (0.97–2.15)	0.070
Discordant	212/1032 (20.5)	38/242 (15.7)	1		1			
Osteoarthritis								
Concordant	966/1032 (93.6)	228/242 (94.2)	1.11 (0.61–2.02)	0.725	1.19 (0.59–2.41)	0.622	1.09 (0.54–2.19)	0.821
Discordant	66/1032 (6.4)	14/242 (5.8)	1		1		1	
Rheumatoid arthritis								
Concordant	998/1032 (96.7)	237/242 (97.9)	1.62 (0.63–4.17)	0.322	1.74 (0.63–4.79)	0.287	1.49 (0.53–4.20)	0.452
Discordant	34/1032 (3.3)	5/242 (2.1)	1		1		1	

* Adjusted for age, sex, income, education, marriage status, physical activity, obesity, smoking habit, frequency of drinking alcohol, and sleep time. † Model 1 plus histories of each disease (osteoporosis, fracture by accident, fracture by falling down, osteoarthritis, and rheumatoid arthritis).“Concordant” means concordant positive–positive or negative–negative result between monozygotic twins or between dizygotic twins, whereas “discordant” means discordant positive and negative results between monozygotic twins or between dizygotic twins.

**Table 3:** Analysis of odds ratios with 95% confidence interval of occurrence of at least one bone disease in monozygotic twins compared to that in dizygotic twins (reference: negative/negative of diseases between twins).

Coincidence of Diseases	Monozygotic Twins	Dizygotic Twins	Odds Ratios (95% Confidence Interval)					
	*n* (%)	*n* (%)	Crude	*p*	Model 1 †	*p*	Model 2 ‡	*p*
Osteoporosis								
Positive–positive	10/1032 (1)	2/242 (0.8)	1.19 (0.26–5.49)	0.819	N/A	N/A	N/A	N/A
Positive–negative	34/1032 (3.3)	4/242 (1.7)	2.03 (0.71–5.78)	0.184	1.94 (0.61–6.11)	0.260	1.81 (0.57–5.71)	0.315
Negative–negative	988/1032 (95.7)	236/242 (97.5)	1		1		1	
Fracture by accident								
Positive–positive	8/1032 (0.8)	6/242 (2.5)	0.30 (0.10–0.87)	0.027 *	0.29 (0.09–0.88)	0.029 *	0.25 (0.08–0.79)	0.018 *
Positive–negative	144/1032 (14)	38/242 (15.7)	0.85 (0.58–1.26)	0.422	0.99 (0.65–1.49)	0.946	0.95 (0.63–1.44)	0.809
Negative–negative	880/1032 (85.3)	198/242 (81.8)	1		1		1	
Fracture by falling down								
Positive–positive	22/1032 (2.1)	4/242 (1.7)	1.38 (0.47–4.05)	0.559	1.67(0.53–5.26)	0.380	1.68 (0.52–5.43)	0.390
Positive–negative	212/1032 (20.5)	38/242 (15.7)	1.40 (0.96–2.04)	0.083	1.42 (0.96–2.09)	0.077	1.41 (0.95–2.08)	0.085
Negative–negative	798/1032 (77.3)	200/242 (82.6)	1		1		1	
Osteoarthritis								
Positive–positive	4/1032 (0.4)	0/242 (0)	N/A	N/A	N/A	N/A	N/A	N/A
Positive–negative	66/1032 (6.4)	14/242 (5.8)	1.12 (0.62–2.03)	0.720	1.18 (0.61–2.26)	0.625	1.10 (0.57–2.11)	0.781
Negative–negative	962/1032 (93.2)	228/242 (94.2)	1		1		1	
Rheumatoid arthritis								
Positive–positive	2/1032 (0.2)	0/242 (0)	N/A	N/A	N/A	N/A	N/A	N/A
Positive–negative	34/1032 (3.3)	5/242 (2.1)	1.62 (0.63–4.18)	0.320	1.76 (0.67–4.64)	0.251	1.52 (0.57–4.05)	0.404
Negative–negative	996/1032 (96.5)	237/242 (97.9)	1		1		1	

* Significance at *p* <0.05. † Adjusted for age, sex, income, education, marriage status, physical activity, obesity, smoking habit, frequency of drinking alcohol, and sleep time. ‡ Model 1 plus histories of each disease (osteoporosis, fracture by accident, fracture by falling down, osteoarthritis, and rheumatoid arthritis).

**Table 4:** Analysis of estimated values of absolute value of difference between the matched twins (reference: absolute value of difference between monozygotic twins).

Difference of Clinical Examination	Monozygotic Twins	Dizygotic Twins	Estimated Values of Absolute Difference between Twin (95% CI)					
	Mean (SD)	Mean (SD)	Crude	*p*	Model 1 †	*p*	Model 2 ‡	*p*
Difference in DEXA T-score	0.5 (1.1)	1.1 (1.7)	0.64 (0.47–0.81)	<0.001 *	0.62 (0.45–0.79)	<0.001 *	0.62 (0.45–0.79)	<0.001 *

Abbreviation: DEXA, dual-energy X-ray absorptiometry; 95% CI, 95% confidence interval; SD, standard deviation. * Significance at *p* < 0.05. † Adjusted for age, sex, income, education, marriage status, physical activity, obesity, smoking habit, frequency of drinking alcohol, and sleep time. ‡ Model 1 plus histories of each disease (osteoporosis, fracture by accident, fracture by falling down, osteoarthritis, and rheumatoid arthritis).

## Data Availability

Restrictions apply to the availability of these data. Data were obtained from the Korean Genome and Epidemiology Study (KoGES) and are available at https://www.nih.go.kr/contents.es?mid=a50401010100#1 (accessed on 1 January 2022).
